# Prediction of Age at Menopause from Assessment of Ovarian Reserve May Be Improved by Using Body Mass Index and Smoking Status

**DOI:** 10.1371/journal.pone.0057005

**Published:** 2013-03-07

**Authors:** Antonio La Marca, Giovanna Sighinolfi, Enrico Papaleo, Angelo Cagnacci, Annibale Volpe, Malcolm J. Faddy

**Affiliations:** 1 Mother-Infant Department, Institute of Obstetrics and Gynecology, University of Modena and Reggio Emilia, Modena, Italy; 2 Centro Natalità, Gynecological-Obstetrics Department, San Raffaele Hospital, Vita-Salute San Raffaele, Milano, Italy; 3 Mathematical Sciences, Queensland University of Technology, Brisbane, Queensland, Australia; University of Valencia, Spain

## Abstract

**Objective:**

Menopause is the consequence of exhaustion of the ovarian follicular pool. AMH, an indirect hormonal marker of ovarian reserve, has been recently proposed as a predictor for age at menopause. Since BMI and smoking status are relevant independent factors associated with age at menopause we evaluated whether a model including all three of these variables could improve AMH-based prediction of age at menopause.

**Methods:**

In the present cohort study, participants were 375 eumenorrheic women aged 19–44 years and a sample of 2,635 Italian menopausal women. AMH values were obtained from the eumenorrheic women.

**Results:**

Regression analysis of the AMH data showed that a quadratic function of age provided a good description of these data plotted on a logarithmic scale, with a distribution of residual deviates that was not normal but showed significant left-skewness. Under the hypothesis that menopause can be predicted by AMH dropping below a critical threshold, a model predicting menopausal age was constructed from the AMH regression model and applied to the data on menopause. With the AMH threshold dependent on the covariates BMI and smoking status, the effects of these covariates were shown to be highly significant.

**Conclusions:**

In the present study we confirmed the good level of conformity between the distributions of observed and AMH-predicted ages at menopause, and showed that using BMI and smoking status as additional variables improves AMH-based prediction of age at menopause.

## Introduction

Age at menopause has relevant implications for female health since late menopause is associated with increased risk of breast cancer [1] and early menopause is associated with increased risk of osteoporosis, cardiovascular disease, early cognitive decline, ovarian cancer, colorectal cancer, respiratory and urogenital disease [2], [3], [4], [5], [6].

More importantly, as women increasingly postpone childbirth, prediction of an early menopause in young women could be of increasing clinical value. The determinants of age at menopause have been investigated in several studies [7], [8] and the most consistent finding is that early age at menopause is associated with smoking and low BMI [8], [9], [10], [11]. Less clear is the relationship between the number of pregnancies and births and the use of hormonal contraception [8].

Since menopause is the consequence of exhaustion of the ovarian follicular pool, recent theories show convincingly that in women of the same age, a larger pool of resting follicles may be associated with a later age at menopause, whereas a smaller pool may be a risk for early menopause [12], [13], [14].

Unfortunately to date there are no diagnostic methods to measure directly the number of primordial follicles in the ovaries of women, while several indirect ovarian reserve markers have been developed and successfully tested [15], [16], [17]. Hormonal (AMH, FSH, inhibin B) and ultrasound (antral follicle count – AFC) markers are associated with antral follicles actually present in the ovaries. However, since the population of antral follicles is related to the number of primordial follicles [12] their determination permits assessment of the extent of the “true” ovarian reserve (the number of non-growing follicles). AMH and AFC have both been shown to have very good and highly significant correlations (R>0.7; *p*-value<0.001) with the number of primordial follicles as determined by modern stereology techniques from histological analysis [18].

AMH has been recently proposed as a good predictor for age at menopause [19], [20], [21], with all studies suggesting that in women of similar age a lower serum AMH level may be indicative of an earlier age at menopause. Without data directly correlating AMH with menopausal age, the methodology in ref.19 was based on joint modelling of data on changing AMH with age and data on menopausal ages, from two independent samples of women, using a hypothesis that AMH falling below a critical threshold is predictive of menopause. Predictions were based only on serum AMH levels and age; however, since BMI and smoking status are relevant independent factors associated with age at menopause, we consider whether similar but more complex modelling including these additional covariates (BMI and smoking) might improve AMH-based predictions of age at menopause.

## Materials and Methods

### Study design and subjects

This cross-sectional study was based on methodology developed in previous studies [19], [22] and involved two independent samples of women. One, a group of 375 healthy, regular cycling, caucasian women aged 19–44 years, was recruited from women requiring preconception counselling or undergoing cervical cancer and breast cancer screening. Inclusion criteria for enrolment were: normal menstrual cycles (length 25–35 days), not pregnant or using hormones or drugs that interfere with the menstrual cycle, no history of hysterectomy, miomectomy, oopherectomy, or any other surgery on their ovaries. Patients included in the study had no known chronic, systemic, metabolic or endocrine disease. All women gave their written informed consent before blood sampling for AMH determination. IRB approval was obtained.

A distribution of ages at menopause was obtained from another sample of 2635 Italian women participating in the GOERM study [23]. This GOERM study was a retrospective study focused on clinical research on menopause for women living in the Italian region of Emilia Romagna and involving four university hospitals (Bologna, Ferrara, Parma and Modena). All women (age at the time of inclusion ranged from 41 to 61 years of age) were menopausal (physiological menopause defined as amenorrhea for more than 12 months); the time since menopause was 2.3±0.02 years (mean ± SEM), and for all women smoking habits and BMI were known, with the latter categorised under-weight (BMI<18.5), normal weight (18.5≤BMI<25), over-weight (25≤BMI<30) and obese (BMI≥30).

### AMH assay

The blood sample for AMH determination was taken when the patients were recruited, independently of the last menstrual cycle. After 12-hour fasting, blood was taken from the cubital vein between 8:00 a.m. and 12:00 noon The blood was centrifuged at 3500 cycles/minute for 10 minutes and the serum was stored in polypropylene tubes at −80°C.

Serum AMH was measured by enzyme-linked immunosorbent assay (ELISA) using the Beckman Coulter, Inc. (Chaska, MN, USA) AMH ELISA kit (Immunotech version, Marseilles, France). The detection limit of the assay was 0.14 ng/ml; imprecision of the assay was 12.3% at 0.2 ng/ml and 5.1% at 15.8 ng/ml. The immunoassay is specific for AMH. No cross-reaction was observed with transforming growth factor-beta.

### Statistical Analysis

Analyses were based on a methodology similar to that used in previous work [19], [22], and involved a two-stage modelling and estimation process using the two data-sets (AMH measurements and menopausal age). First, a robust regression analysis of the logged AMH data as the response (or dependent variable) using age, BMI and smoking status as covariates (or independent variables) was carried out by maximum likelihood, using modelling with a more general (longer-tailed than normal) skew-*t* residual distribution as described in [24]. The estimated regression equation and probability distribution of residual deviates established a model for age-related change in AMH, from which age-dependent AMH-percentiles (5%, 10%, 25%, 50%, 75%, 90% and 95%) could be estimated.

The second stage uses the hypothesis [19] that occurrence of menopause can be predicted by AMH falling below a critical threshold level, which provides a link between the two data-sets whereby menopause occurring before age *y* (say) corresponds to AMH at age *y* being below this threshold. This enables a probability distribution of menopausal ages to be determined from the equation:
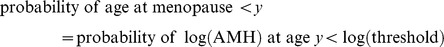
using the previously estimated regression equation for the mean of log(AMH) at age *y*. To allow for excess inter- and intra-cycle variation in AMH from individual women [25] not contributing to varying fertility between women, another skew-*t* probability distribution [24] was used to describe the variation of log(AMH) here. With log(threshold) a linear function of BMI and smoking status (as in the usual regression context) probabilities on the right hand side of the above equation can be determined. This formulates a model for analysis of the GOERM data using maximum likelihood estimation, where menopausal age is the response and BMI and smoking status are covariates. Finally, percentiles of menopausal age can be calculated from the estimated BMI and smoking specific probability distributions of menopausal age, similar to those for AMH.

Prediction of menopausal age for individual women follows a similar two stage process. First, the woman's AMH level and age is located within age-dependent AMH percentiles (less than 5%, between 5% and 10%, etc.), then her predicted age at menopause can be inferred from similar percentiles of menopausal age.

## Results

Characteristics of patients included in the study are reported in **Table 1**. In the AMH cohort the percentage of smokers was significantly higher than in the GOERM study. BMI was significantly higher for women from GOERM than for those in the AMH cohort, probably because women in the latter group were younger than those in the former group, providing a *prima facie* case for some allowance of these covariates (smoking and BMI) in the analysis. In the GOERM data-set (*n* = 2635), the mean age at which women reported menopause was 49.4±0.8 years (mean ± SEM).

**Table 1:** Characteristics of patients in the AMH and GOERM cohorts.

	AMH	GOERM	Significance of difference
Age at inclusion(mean ± SEM)	35.3±0.2	52.5±0.1	*p*-value<0.05
Current or past smoker (%)	33.5	27.6	*p*-value<0.05
BMI (mean ± SD)	23.2±4.2	26±4.6	*p*-value<0.05

The regression analysis of the AMH data showed that age (*p*-value<0.001) and age^2^ (*p*-value≈0.03) were significant predictors while BMI and smoking status were not (*p*-values>0.20), giving (*cf.* the preferred model in [26]): 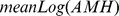 = 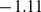
+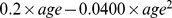


**Figure 1:**
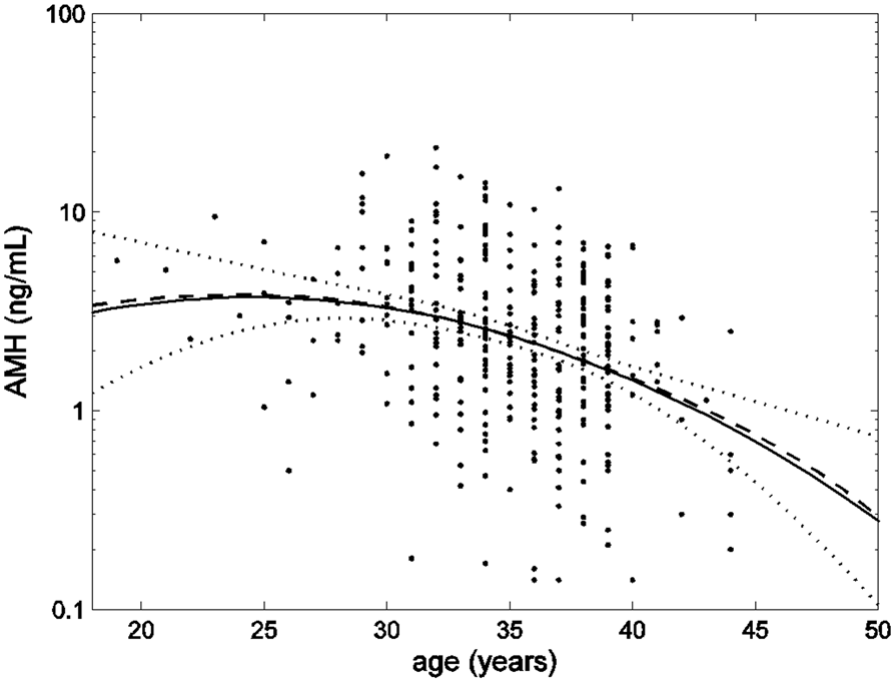
AMH data (dots) plotted on a logarithmic scale with smoothed mean (dashed line) similar to ref.19, and an estimate of the mean as a quadratic function of age (solid line) with upper and lower 95% confidence limits (dotted lines).

This quadratic function of age was very close to an estimate of the mean obtained by smoothing the raw observations as in [19] shown in **Figure 1**, with AMH declining from a peak at around age 25 years. Additional terms involving age^3^ and age^4^ were not at all significant (*p*-values>0.50), and BMI and smoking status also had no significant effects on the rates of change with age (*p*-values>0.17).

**Figure 2:**
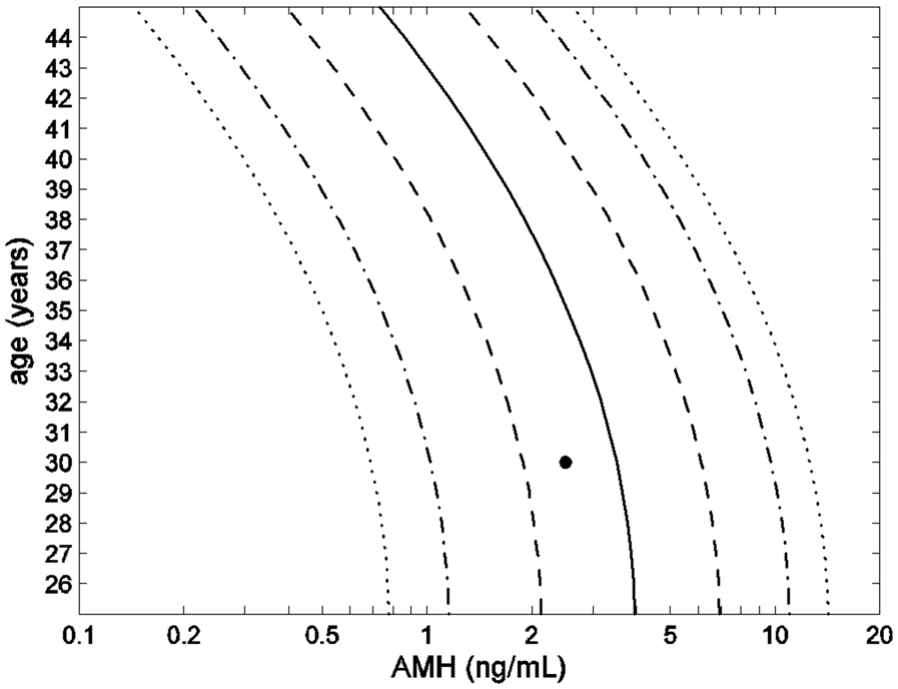
Age-dependent AMH-percentiles: 5% & 95% (dotted lines), 10% & 90% (dash-dot-dash lines), 25% & 75% (dashed lines), and 50% or median (solid line), with • denoting AMH level 2.5 ng/mL at age 30 years.

The distribution of the residual deviates (differences between data values and estimated mean) was significantly (*p*-value<0.01) non-normal showing left-skewness (relatively more subjects had AMH levels above the estimated mean than below it) and was adequately described by a skew-*t* distribution (goodness of fit statistic 373.8 on 369 degrees of freedom). Estimated AMH-percentiles from the resulting model are shown in **Figure 2**, with the age range restricted to 25–45 years where the mean is relatively well estimated (**Figure 1**). Notice that the estimated median (50-percentile) is slightly larger than the estimated mean (**Figure 1**), reflecting the left-skewness of the residual distribution. **Figure 2** indicates that an AMH level of 2.5 ng/mL from a 30-year old woman, for example, would be between the 25- and 50-percentiles.

**Figure 3:**
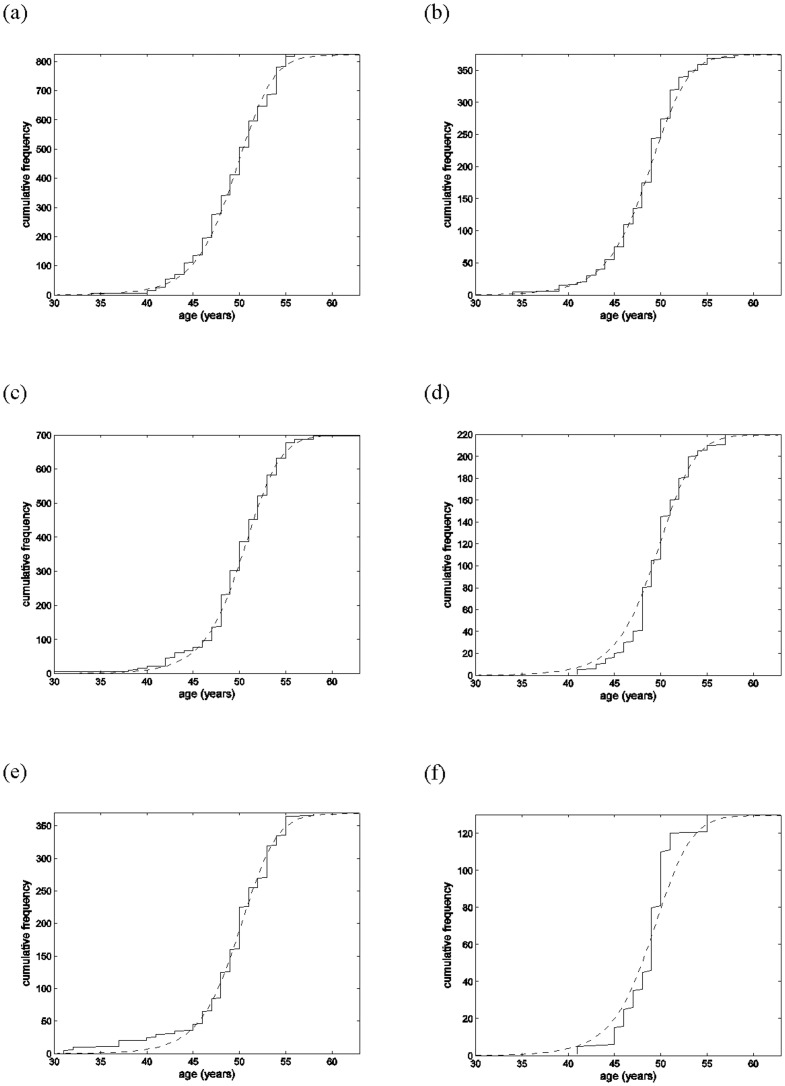
(a)–(f): comparisons of cumulative frequency distributions of age at menopause from the GOERM study (solid lines) and model-based predictions from AMH falling below a critical threshold (dashed lines) for different BMI categories and smoking status, showing quite good concordance between these. a): normal weight non-smokers ; (b): normal weight current or past smokers ; (c): over-weight non-smokers ; (d): over-weight current or past smokers ; (e): obese non-smokers; (f): obese current or past smokers.

The subsequent analysis of the GOERM data using the model for menopausal ages derived from AMH falling below a critical threshold dependent on BMI and smoking status (combined additively in the log-threshold) showed significant effects of these covariates (*p*-values<0.001). Some comparisons of the observed and modelled cumulative frequency distributions of menopausal age for different BMI categories and smoking status are displayed in **Figure 3**, showing quite good concordance between these – particularly for the normal and over-weight BMI categories which covered most (80%) of the data. (No meaningful comparisons could be made for the under-weight BMI category as only 15 observations were in this category.) The residual variance of log(AMH) from this analysis was, significantly (*p*-value<0.001) lower than that from the earlier regression analysis of log(AMH) on age, by an estimated factor of 0.72 (95% confidence interval 0.62–0.85) consistent with some excess inter- and intra-cycle variation in the AMH data. The estimated thresholds below which AMH predicts menopause are shown in **Table 2**, for non-smokers and current or past smokers, and the different BMI categories.

**Table 2:** Estimated thresholds below which AMH predicts menopause (with 95% confidence intervals in brackets) according to smoking status and BMI categories.

BMI	non-smoker	current or past smoker
Under-weight	0.45 ng/mL (0.21, 0.97)	0.53 ng/mL (0.27, 1.07)
Normal weight	0.31 ng/mL (0.13, 0.74)	0.36 ng/mL (0.16, 0.81)
Over weight	0.25 ng/mL (0.09, 0.69)	0.30 ng/mL (0.12, 0.75)
Obese	0.28 ng/mL (0.11, 0.71)	0.33 ng/mL (0.14, 0.78)

From probability distributions of menopausal ages based on predictions from AMH falling below these thresholds, percentiles for non-smokers and current or past smokers, and the different BMI categories, were estimated and are shown in **Table 3**. And in **Table 4** are the corresponding percentiles of menopausal age from similar modelling based on the critical AMH threshold having no dependence on BMI and smoking status (*cf.* [19]), where it can be seen that these percentiles are quite similar to those in **Table 3** for normal weight non-smokers which was the modal category (with almost one-third of the data). These will give some indication of likely age at menopause from the corresponding percentile band where a woman's age and AMH level was located in **Figure 2**; for example, a woman with AMH between the 25- and 50-percentiles who was of normal weight and a non-smoker or over-weight and a current or former smoker could be expected to experience menopause between 47 and 49.5 years of age (**Table 3**). But if she were an overweight non-smoker then she could expect almost another year before menopause, and about one year less if a normal weight current or former smoker.

**Table 3:** Estimated percentiles of the probability distributions of age at menopause (± standard errors in brackets) predicted by AMH falling below critical thresholds according to smoking status and BMI categories.

AMH critical thresholds	Body weight	Predicted age at menopause (± standard error)	
		Non-smoker	Current or past smoker
5°	Under-weight	39 (1.6)	37.6 (1.8)
	Normal weight	42.1 (0.2)	40.9 (0.3)
	Over-weight	43.4 (0.3)	42.2 (0.3)
	Obese	42.7 (0.3)	41.5 (0.4)
10°	Under-weight	41.6 (1.3)	40.3 (1.5)
	Normal weight	44.2 (0.2)	43.1 (0.2)
	Over-weight	45.3 (0.2)	44.3 (0.3)
	Obese	44.7 (0.2)	43.6 (0.8)
25°	Under-weight	44.7 (1.1)	43.6 (1.2)
	Normal weight	47 (0.1)	46 (0.2)
	Over-weight	47.9 (0.1)	47 (0.2)
	Obese	47.4 (0.2)	46.5 (0.2)
50°	Under-weight	47.5 (1)	46.5 (1)
	Normal weight	49.5 (0.1)	48.6 (0.2)
	Over-weight	50.4 (0.1)	49.5 (0.2)
	Obese	49.9 (0.2)	49 (0.2)
75°	Under-weight	49.9 (0.9)	49 (0.9)
	Normal weight	51.7 (0.1)	50.9 (0.2)
	Over-weight	52.5 (0.1)	51.7 (0.2)
	Obese	52.1 (0.2)	51.3 (0.2)
90°	Under-weight	51.9 (0.8)	51.1 (0.9)
	Normal weight	53.6 (0.1)	52.9 (0.2)
	Over-weight	54.4 (0.1)	53.6 (0.2)
	Obese	54 (0.2)	53.2 (0.2)
95°	Under-weight	53.1 (0.8)	52.4 (0.9)
	Normal weight	54.8 (0.1)	54.1 (0.2)
	Over-weight	55.5 (0.1)	54.8 (0.2)
	Obese	55.1 (0.2)	54.4 (0.2)

**Table 4:** Estimated percentiles of the probability distribution of age at menopause (± standard errors in brackets) predicted by AMH falling below a critical threshold ignoring smoking status and BMI categories.

AMH critical threshold	Predicted age at menopause (± standard error)
5°	42.3 (0.2)
10°	44.3 (0.1)
25°	47.1 (0.1)
50°	49.6 (0.1)
75°	51.8 (0.1)
90°	53.8 (0.1)
95°	55 (0.1)

## Discussion

Age at menopause varies widely in the female population; indeed, the range for age at menopause is commonly believed to be between 40 and 60 years [27]. The wide variability in age at menopause is assumed to be the reflection of the high variability in ovarian reserve for women of similar ages. According to recent models describing the rate of follicular decline with aging [13], [14], the pool of primordial follicles does vary widely in women of similar ages. For example, at the age of 35 y the estimated range of resting follicles in the ovary is between 19000 and 135000 [14]. Such high variability is then reflected in a higher variability of the age at which primordial follicles will be exhausted and women enter menopause.

Hence any biomarker indicating the number of resting follicles may contribute to improvement in the prediction of menopause based on age alone. AMH, a glycoprotein produced and secreted by primary and preantral follicles, has been proposed as a reliable measure of ovarian reserve. Indeed, serum AMH levels decrease throughout reproductive life and are undetectable several years before physiological menopause or following ovarian surgery [16]. With respect to other known markers, AMH seems to better reflect the continuous decline of the follicular pool with age [20]. Some years ago the possibility that AMH may permit prediction of age at menopause was demonstrated in a cross sectional study based on 144 women [19]. In that study the predicted age at menopause ranged from less than 41 years to more than 56 years according to the age specific percentiles of AMH, whereby the lower age specific AMH value, the lower the predicted age at menopause. Subsequently the hypothesis has been confirmed by a 6-year longitudinal study performed on 147 over 40-year old women [28], which reported that a basal AMH lower than 0.39 ng/mL may predict occurrence of menopause in the next 6 years with a positive predictive value of 0.9 and a negative predictive value of 0.76. Both these groups of researchers recently published well-designed longitudinal studies validating this hypothesis [21], [29]. In particular, in the Dutch study 257 women (aged 21–46 y) were followed for 11 years; 19% of women experienced menopause in this time frame and basal AMH was significantly related to time to menopause and showed a good proportion of correct predictions [29].

In the present study we have confirmed the good level of conformity between the distribution of observed age at menopause and predictions based on falling AMH levels. This enables clinicians to have a useful assessment of the remaining reproductive life span, particularly for women in the age range 30–40 years where the AMH-percentiles are quite well estimated.

The main contribution of this study has been that other variables were used in addition to AMH in order to improve prediction of age at menopause, namely BMI and smoking status. A large (*n* = 31000) Italian retrospective study [8] showed that low BMI and smoking were both independently associated with early age at menopause. Other retrospective and prospective studies confirmed the positive correlation existing between BMI and age at menopause [10], [11], [30]. Similarly, smoking has been repeatedly reported as being associated with an earlier age at menopause [8], [9], [10], [11]. Our study confirms the influence of BMI and smoking status on AMH-based prediction of age at menopause, by showing that the threshold below which AMH predicts menopause varies significantly with these covariates. However, estimates of the actual thresholds were rather imprecise with up to a 7-fold difference between the upper and lower 95% confidence limits, and the latter below the detection limit of the AMH assay for some covariate values (**Table 2**). This is due largely to similar imprecision in the estimated mean AMH level at the mean age of menopause (49.4 y) – see **Figure 1**.

Despite this imprecision, the modelled distributions of age at menopause were quite precisely estimated (due to inter-dependence between the AMH regression and AMH threshold components of the modelling, whereby differences between these can be better estimated than the individual components). The variation in AMH necessary to describe the variation in menopausal ages from predictions based on AMH falling below critical thresholds was significantly less than the residual variation from the regression model of AMH and age, and consistent with excess inter-and intra-cycle variation affecting these AMH data whereby 76% of the age-adjusted AMH variance can be attributed to variation between women (with 11% and 13% of the variance due to inter- and intra-cycle variation, respectively, from individual women [25]).

According to predictions without reference to BMI and smoking (**Table 4** and ref.19) a woman with AMH at the 10% age-specific percentile level would be predicted to experience menopause at about 44 years of age, while predictions range from 40.3 to 45.3 years if allowance is made for BMI and smoking status (**Table 3**). Menopausal age will generally be lower for low BMI and for current or past smokers (**Table 3**).

The combination of BMI and smoking status with AMH for prediction of age at menopause is quite practicable since these two covariates were not significantly associated with AMH levels. While AMH seems to be lower in women with high BMI, any association did not reach statistical significance when allowance was made for age (i.e., older women tend to have lower AMH and higher BMI than younger women). Several studies have reported a negative association between smoking and AMH levels [31] while others have reported non significant relationships [16], [32]. The largest cross-sectional study reporting on AMH, smoking and BMI and based on 416 women from the general population [33] found no age-independent correlation between AMH and both these covariates.

In conclusion, the present study confirms that serum AMH levels enable the prediction of a woman's reproductive life span, and that that such prediction may be refined by other easily acquirable information on BMI and smoking status that are associated with age at menopause.
